# Vegetative Propagule Pressure and Water Depth Affect Biomass and Evenness of Submerged Macrophyte Communities

**DOI:** 10.1371/journal.pone.0142586

**Published:** 2015-11-11

**Authors:** Hong-Li Li, Yong-Yang Wang, Qian Zhang, Pu Wang, Ming-Xiang Zhang, Fei-Hai Yu

**Affiliations:** School of Nature Conservation, Beijing Forestry University, Beijing, 100083, China; Shandong University, CHINA

## Abstract

Vegetative propagule pressure may affect the establishment and structure of aquatic plant communities that are commonly dominated by plants capable of clonal growth. We experimentally constructed aquatic communities consisting of four submerged macrophytes (*Hydrilla verticillata*, *Ceratophyllum demersum*, *Elodea nuttallii* and *Myriophyllum spicatum*) with three levels of vegetative propagule pressure (4, 8 and 16 shoot fragments for communities in each pot) and two levels of water depth (30 cm and 70 cm). Increasing vegetative propagule pressure and decreasing water level significantly increased the growth of the submerged macrophyte communities, suggesting that propagule pressure and water depth should be considered when utilizing vegetative propagules to re-establish submerged macrophyte communities in degraded aquatic ecosystems. However, increasing vegetative propagule pressure and decreasing water level significantly decreased evenness of the submerged macrophyte communities because they markedly increased the dominance of *H*. *verticillata* and *E*. *nuttallii*, but had little impact on that of *C*. *demersum* and *M*. *spicatum*. Thus, effects of vegetative propagule pressure and water depth are species-specific and increasing vegetative propagule pressure under lower water level can facilitate the establishment success of submerged macrophyte communities.

## Introduction

Submerged macrophyte communities are vital components of many aquatic ecosystems [1] because they can purify water and provide food and habitat for aquatic fauna [2, 3]. The successful establishment of submerged macrophyte communities is a critical step to restore functions of degraded aquatic ecosystems [4, 5]. Thus factors determining the success of submerged macrophyte communities warrant extensive studies [6, 7].

Propagule pressure, i.e. the availability of sexual or asexual (vegetative) propagules, is an important factor determining the establishment success of plant populations or communities, especially during the early establishment phase [8–11]. Studies have indeed shown that greater seed availability significantly contributed to the establishment success of both invasive and native plant species in grasslands and that greater number of ramets increased biomass of an invasive plant [8, 12]. Most submerged macrophytes have the ability to propagate vegetatively [13, 14]. They can sustain and expand their original populations and also establish new populations through vegetative progagules [14, 15]. Consequently, vegetative propagule pressure may be a key determinant of the establishment, growth and spread of submerged macrophyte communities [16].

Water depth is a crucial factor for the functional stability of aquatic ecosystems [7, 17]. Managing water depth is an efficient way for lake restoration, macrophyte community construction and other ecological projects [18–20]. This is partly because water depth can greatly impact the establishment, growth and spread of submerged macrophyte communities by changing underwater light, nutrients and oxygen content [21–23]. The depth at which submerged macrophytes can grow is often related to water clarity. In shallow, clear water bodies, submerged macrophytes are responsible for a main part of total primary production. In deeper lakes, the importance and abundance of submerged macrophytes depend on a number of factors; but, above all, there is a strong relation between maximum growing depth and light conditions [22, 24]. Generally, a certain level of water depth is conducive to the maintenance of stable macrophyte communities [25], whereas excessive high or low water depth is detrimental to the stability of submerged macrophyte communities [26, 27]. Many submerged macrophytes can, to some degree, tolerate different water depths by altering their morphology and physiology, but the degree of tolerance differs among species [7, 28–30]. Thus, water depth may greatly affect the establishment success and also species composition of submerged macrophyte communities.

We designed a microcosm experiment to test effects of vegetative propagule pressure and water depth on the growth and species composition of submerged macrophyte communities during the early stage of their establishment. We varied number of initial shoot fragments of the submerged macrophytes to represent different levels of vegetative progagule pressure because shoot fragments are commonly used for revegetation of submerged macrophyte communities [31, 32]. We hypothesized that increasing vegetative propagule pressure would increase the establishment success of submerged macrophyte communities. Since effects of vegetative pressure may differ among species, we also hypothesized that increasing propagule pressure would change species composition of submerged macrophyte communities. Furthermore, compared to high water depth, relatively low water depth may facilitate the establishment of submerged macrophyte communities because of increased light availability. Thus we predicated that (1) high vegetative propagule pressure and low water depth would increase the growth of submerged macrophyte communities, (2) effects of vegetative propagule pressure and water depth would differ among the submerged macrophytes, and (3) varying propgagule pressure and water level would change species composition of the submerged macrophyte communities.

## Materials and Methods

### Experimental communities and component species

The experimental communities consisted of four submerged macrophytes, i.e. *Hydrilla verticillata* (L.f.) Royle (Hydrocharitaceae), *Ceratophyllum demersum* L. (Ceratophyllaceae), *Elodea nuttallii* (Planch.) St John (Hydrocharitaceae), and *Myriophyllum spicatum* L. (Haloragaceae) [29]. All of the four species are perennial, aquatic clonal plants [33, 34] and their shoot fragments can easily develop into a whole plant. We chose these four species because they can coexist in lakes (Yong-Yang Wang, personal observation), are easy to obtain, and are commonly used for restoration of degraded aquatic ecosystems in China.

*H*. *verticillata* grows in various habitats such as lakes, ditches and rivers. Shoots of this macrophyte are fragile and can be easily broken by external forces such as water current, weeding and rowing [35]. In Beijing, China, blossoms occur from August to September [36], and fruits occur from September to October. *C*. *demersum* is a cosmopolitan species and commonly grows in shallow lakes and pools where water depth is less than 1.5 m. This species blossoms from June to July, and fruits from August to September [29]. *E*. *nuttallii* was introduced to China in the 1980s from Japan [37]. It can grow in lakes and pools, and blossom from July to September. *M*. *spicatum* has lobe linear leaves, and can adapt to a wide variety of water environments, blossoming from June to August.

### Sampling and experimental design

In late June 2012, shoot fragments of the four submerged macrophytes were collected in the lakes of Winter Place (40°00'15.96" N; 116°18'11.26" E) and Olympic Green (40°00'43.50" N; 116°23'07.31" E) in Beijing. The collections of the samples were allowed by the administrative department of the both parks. Seventy mature shoot fragments with an apex were selected for each species. Each fragment was cut to 13 cm long with the apex and with about 25 nodes in *E*. *nuttallii* and about 13 nodes in the other three species. All side branches were removed.

The experiment was conducted outdoors in the Cuihu National Urban Wetland Park in the suburb area of Beijing, China on 25 August 2012. The experiment took a split-plot design with two levels of water depth (30 cm and 70 cm deep) as the main plot factor and three levels of propagule pressure (low, medium and high propagule pressure) as the sub-plot factor. The 30 cm and 70 cm water depth were defined as low and high water depth. We chose these two water depths because they represent two of the most common water depths that many submerge macrophytes can grow. In the low, medium and high propagule pressure treatment, each community was initially composed of 1, 2 and 4 shoot fragments of each of the four submerged macrophytes, respectively. Thus each experimental community initially consisted of 4, 8 and 16 shoot fragments, corresponding to about 157, 314 and 628 shoots m^-2^. Such shoot densities are within the range of the shoot densities of submerged macrophyte communities in natural conditions.

Shoot fragments comprising each experimental community were planted in a plastic pot (18 cm in diameter × 12 cm in depth) filled with 12-cm-deep river sediment collected in the park (Organic matter: 14.0 ± 1.00 g kg^-1^, TN: 0.8 ± 0.07 g kg^-1^, TP: 6.7 ± 0.04 g kg^-1^, mean ± SE, n = 3) and placed inside a mesh container (19 cm in diameter × 100 cm in height). The mesh container (with the pot and plants) was submerged in one of the ten plastic tanks (150 cm in diameter × 82 cm in depth) full of water collected from the lake of the park (TN: 0.74 ± 0.03 mg L^-1^, TP: 0.0015±0.0004 mg L^-1^, n = 3) (United intelligence, Beijing, China). In five tanks the water depth was adjusted to 30 cm above the sediment surface in pots, and in the other five it was adjusted to 70 cm. Each treatment had five replicates and each replicate was placed in a tank. The water in each tank was replaced once in the middle of the experiment. Light intensity in the water column (30 cm and 70 cm water depth) was measured by a Li-COR UWQ-4341 sensor. The light intensity at 30 cm and 70 cm depth under the water was 313.4 ± 9.71 and 77.2 ± 4.64 μmol m^-2^ s^-1^ (mean ± SE, n = 3) respectively, at noon on 19 September 2012. Mesh containers confined communities to prevent interference within the tanks.

### Harvest and measurements

The experiment lasted seven weeks and ended on 13 October 2012. All the surviving submerged macrophytes (including roots) were carefully harvested and sorted into species. Number of shoot nodes and total shoot length are measures of potential clonal growth and local vegetative spread, because every single node can potentially develop into a new plant. These two measures were determined during the harvest. As the plants were easily broken into numerous shoot fragments during harvest, it was impossible to count node number and measure shoot length for all shoot fragments. We randomly selected five shoot fragments of each species from each experimental community to count their number of nodes and measure their shoot length. Biomass of these samples was measured separately. We then measured biomass of the remaining parts of each species in each community. For biomass measurements, the plants were oven-dried at 80°C for 48 h and weighed.

### Data analyses

For each species in each community (container), we calculated number of nodes per unit mass and shoot length per unit mass based on the measurements (dry biomass, number of nodes and shoot length) on the five selected shoots. Total number of nodes and total shoot length for each species in each community were derived by multiplying total biomass by number of nodes or shoot length per unit mass. Biomass of a community is the sum of the biomass values of the four submerged macrophytes. Similarly, we calculated number of nodes and shoot length of the community (S1 Table).

To measure species diversity of the community, we calculated Pielou evenness index (J) as J = -∑P_*i*_ ln(P_*i*_)/ln(S), where *i* = 1, 2… S, P_*i*_ is biomass of species *i* divided by the sum of biomass of all the species in the community, and S is the number of species in the community [38].

We obtained total biomass, total number of nodes and total shoot length for each species and for each community (each container). We also calculated biomass, number of nodes and shoot length per individual plant as total biomass, total number of nodes and total shoot length for each species divided by the number of individual plants of each species (i.e. 1, 2 and 4 fragments of each of the four submerged macrophytes, respectively).

All data were checked for homoscedasticity and normality. For species level, we transformed values of total biomass, total node number and total shoot length of *C*. *demersum* and *M*. *spicatum* to square root. For individual plants, we transformed biomass of *H*. *verticillata*, node number and shoot length of *C*. *demersum*, biomass and node number of *E*. *nuttallii*, and biomass, node number and shoot length of *M*. *spicatum* to square root.

At community level, we used split-plot ANOVA to test the effects of propagule pressure (low, medium and high) and water depth (30 cm and 70 cm) on biomass, number of nodes, shoot length and evenness of the communities. At species level, we also employed split-plot ANOVA to test the effects of propagule pressure and water depth on total biomass, number of nodes and shoot length of the submerged macrophytes species in the community and biomass, number of nodes and shoot length per individual plant for each of the component species except *M*. *spicatum*. Since all *M*. *spicatum* plants died at the high water depth, we only tested the effects of propagule pressure at the low water depth by one-way ANOVA. All analyses were conducted with SPSS 20.0 software (SPSS, Chicago, IL, USA).

## Results

### Effects at community level

**Fig 1:**
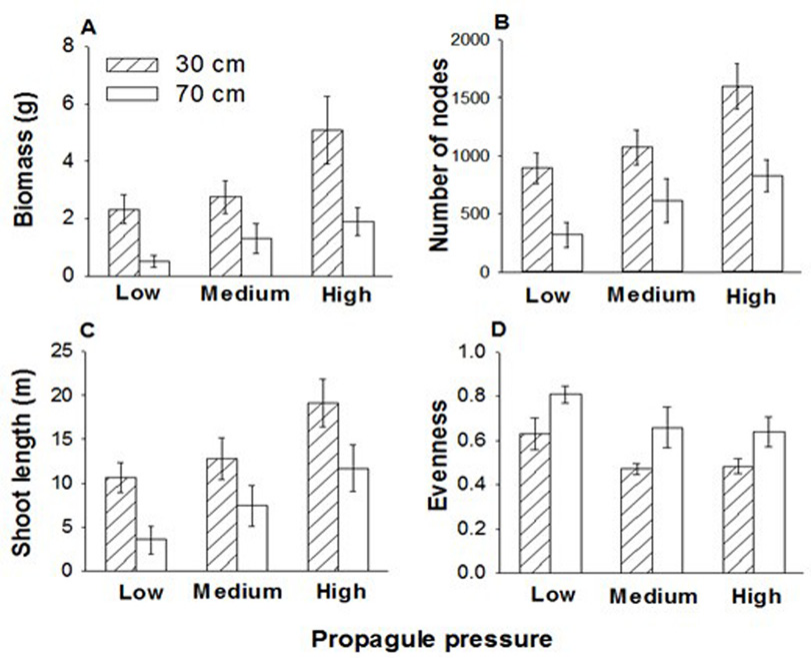
Effects of propagule pressure and water depth on the growth and evenness of the submerged macrophyte communities. Mean values (± SE) are shown.

Vegetative propagule pressure and water depth significantly affected total biomass, node number, shoot length and evenness of the submerged macrophyte communities (Table 1). Total biomass, number of nodes and shoot length of the communities increased significantly with increasing propagule pressure, and when water depth was low (30 cm; Table 1; Fig 1A–1C). However, evenness of the communities significantly decreased with increasing propagule pressure and with decreasing water depth (Table 1, Fig 1D). There was no significant interaction effect of propagule pressure and water depth on any of the three growth measures or evenness of the communities (Table 1, Fig 1).

**Table 1:** Effects of propagule pressure and water depth on the growth and evenness of the submerged communities.

	Propagule pressure (P)		Water depth (W)		P × W	
	*F*_2,16_	*P*	*F*_1,8_	*P*	*F*_2,16_	*P*
Biomass	51.64	<0.001	7.56	0.025	1.14	0.345
Number of nodes	27.62	<0.001	10.47	0.012	1.06	0.369
Shoot length	29.27	<0.001	5.63	0.045	0.11	0.899
Evenness	4.91	0.022	11.85	0.009	0.04	0.961

The given are *F*, *P* and degree of freedom based on split-plot ANOVA.

### Effects at species level

**Fig 2:**
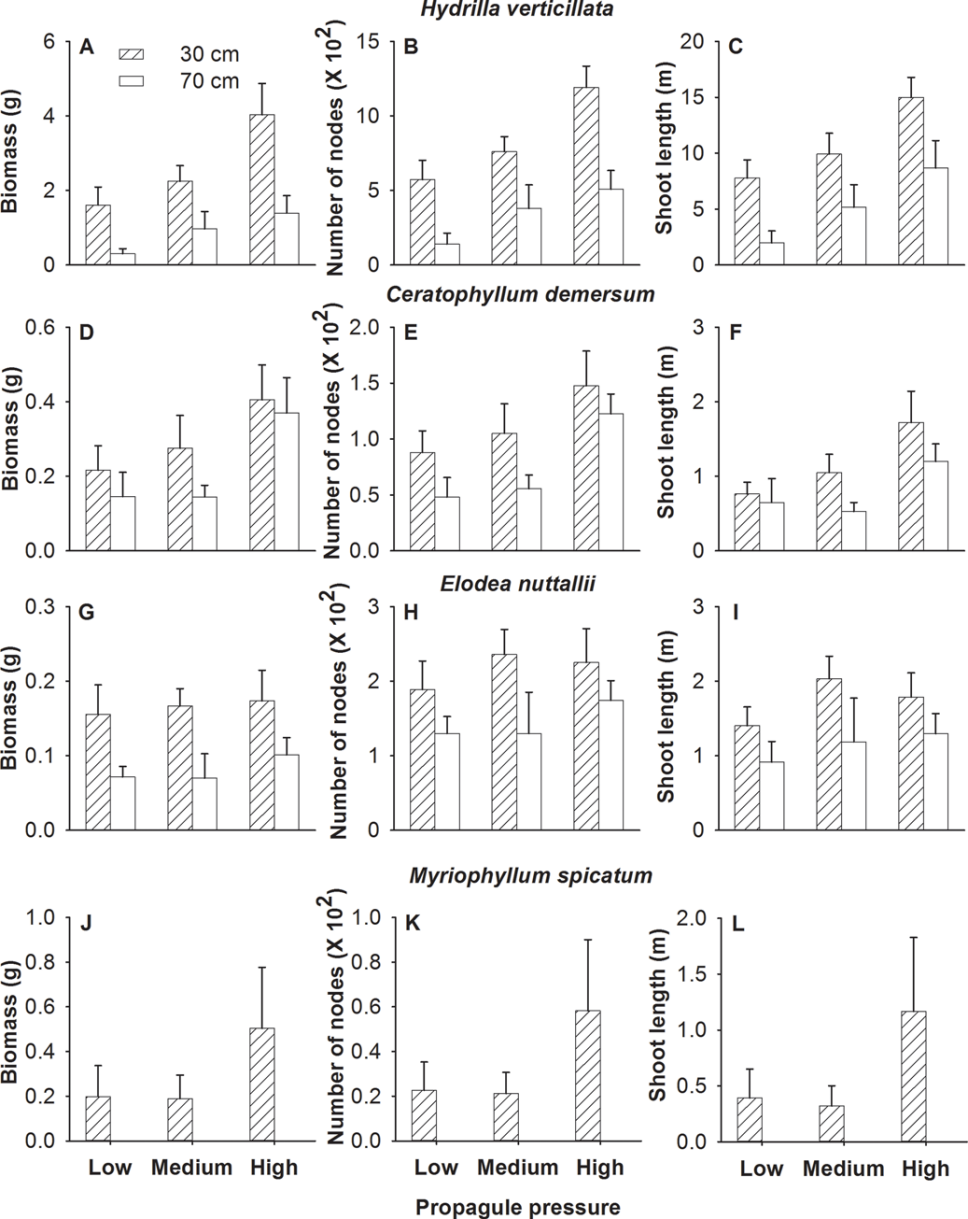
Effects of propagule pressure and water depth on the growth of each of the four submerged macrophytes. Mean values (± SE) are shown.

Increasing propagule pressure significantly or marginally significantly (*P*<0.1) increased total biomass, node number and shoot length of *H*. *verticillata* (Table 2, Fig 2A–2C) and *C*. *demersum* (Table 2, Fig 2D–2F). However, propagule pressure did not affect the growth measures of *E*. *nuttallii* (Table 2, Fig 2G–2I) or *M*. *spicatum* (Table 2, Fig 2J–2L). Increasing water depth significantly decreased total biomass, number of nodes and shoot length of *H*. *verticillata* (Table 2, Fig 2A–2C), number of nodes of *C*. *demersum* and biomass of *E*. *nuttallii* (Table 2, Fig 2E and 2G). No plants of *M*. *spicatum* survived at the 70 cm water depth, but more than 60% survived at the 30 cm water depth (Fig 2J–2L).

**Table 2:** Effects of propagule pressure and water depth on the growth of each of the four submerged macrophytes.

	Propagule pressure (P)		Water depth (W)		P × W	
	*F*_2,16_	*P*	*F*_1,8_	*P*	*F*_2,16_	*P*
(A) *Hydrilla verticillata*						
Biomass	26.29	<0.001	6.88	0.031	5.07	0.020
Number of nodes	22.36	<0.001	10.34	0.012	2.40	0.123
Shoot length	23.81	<0.001	5.77	0.043	0.31	0.739
(B) *Ceratophyllum demersum*						
Biomass^1^	3.52	0.054	2.69	0.140	0.58	0.573
Number of nodes^1^	4.74	0.024	4.60	0.024	1.43	0.268
Shoot length^1^	4.35	0.031	0.77	0.405	1.57	0.239
(C) *Elodea nuttallii*						
Biomass	0.59	0.567	6.19	0.038	0.14	0.871
Number of nodes	0.76	0.484	3.39	0.103	0.41	0.670
Shoot length	1.11	0.355	3.39	0.103	0.20	0.822
(D) *Myriophyllum spicatum* ^2^						
Biomass^1^	0.41	0.670				
Number of nodes^1^	0.38	0.692				
Shoot length^1^	0.54	0.598				

The given are *F*, *P* and degree of freedom based on split-plot ANOVA.

^1^ Data were transformed to square root before analysis.

^2^For *M*. *spicatum*, only the effect of propagule pressure was tested because all *M*. *spicatum* plants at high water depth died.

**Fig 3:**
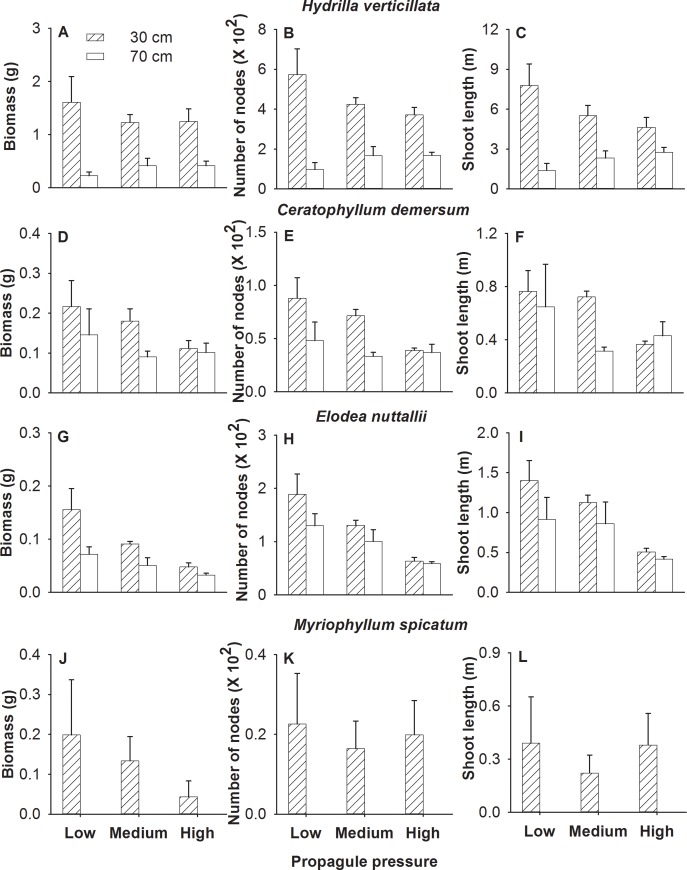
Effects of propagule pressure and water depth on the per individual growth of each species of the four submerged macrophytes. Mean values (± SE) are shown.

Increasing propagule pressure significantly decreased number of nodes and shoot length per individual shoot fragment of *H*. *verticillata* (Table 3, Fig 3B and 3C), and biomass, number of nodes and shoot length per individual fragment of *E*. *nuttallii* (Table 3, Fig 3G–3I). Increasing water depth significantly decreased all three growth measures per individual fragment of *H*. *verticillata*, and such an effect decreased with increasing propagule pressure (Table 3, Fig 3A–3C). Increasing water depth also decreased biomass per individual fragment of *E*. *nuttallii* (Table 3, Fig 3G).

**Table 3:** Effects of propagule pressure and water depth on the growth per individual plant for each of the four submerged macrophytes

	Propagule pressure (P)		Water depth (W)		P × W	
	*F*_2,16_	*P*	*F*_1,8_	*P*	*F*_2,16_	*P*
(A) *Hydrilla verticillata*						
Biomass per individual plant^1^	1.16	0.338	7.84	0.023	3.48	0.055
Number of nodes per individual plant	4.16	0.035	8.96	0.017	4.25	0.033
Shoot length per individual plant	4.92	0.022	6.73	0.032	6.57	0.008
(B) *Ceratophyllum demersum*						
Biomass per individual plant	1.80	0.197	2.48	0.154	0.41	0.668
Number of nodes per individual plant^1^	2.19	0.145	4.77	0.060	1.46	0.261
Shoot length per individual plant^1^	2.39	0.123	0.49	0.505	0.66	0.532
(C) *Elodea nuttallii*						
Biomass per individual plant^1^	12.90	<0.001	5.98	0.040	2.15	0.149
Number of nodes per individual plant^1^	14.16	<0.001	3.52	0.098	1.12	0.351
Shoot length per individual plant	8.58	0.003	3.01	0.121	0.54	0.594
(D) *Myriophyllum spicatum* ^2^						
Biomass per individual plant^1^	0.07	0.934				
Number of nodes per individual plant^1^	0.12	0.888				
Shoot length per individual plant^1^	0.14	0.868				

The given are *F*, *P* and degree of freedom based on split-plot ANOVA.

^1^ Data were transformed to square root before analysis.

^2^ For *M*. *spicatum*, only the effect of propagule pressure was tested because all *M*. *spicatum* plants at high water depth died.

## Discussion

Increasing vegetative propagule pressure increased the establishment success of the submerged macrophyte communities as indicated by their increased growth. Similarly, previous studies showed that increasing propagule pressure could increase the invasion success of some invasive plant species [8, 39]. Thus progagule pressure is a key determinant for successfully establishing plant populations or communities. The results also suggests that propagule pressure should be considered when utilizing vegetative progagules to re-establish submerged macrophyte communities in degraded aquatic ecosystems, especially during the early stage.

Increasing vegetative propagule pressure, however, significantly decreased evenness of the submerged macrophyte communities, because it markedly increased the growth of *H*. *verticillata* and *C*. *demersum*, but has little impact on the growth of the other two species (*E*. *nuttallii* and *M*. *spicatum*). It is widely recognized that increasing propagule pressure may dramatically increase competition intensity among individual plants, greatly affecting size hierarchy of populations and species evenness of communities [40]. Interactions between macrophytes then gradually change, regulating the species growth [41]. In the present study, increasing vegetative propagule pressure also increased competition among species, as indicated by the decreased growth of the dominant species *H*. *verticillata* and the less dominant *E*. *nuttallii* individual plants. Conversely, the competitive effect on *C*. *demersum* was not significantly affected by vegetative propagule pressure. Species-specific responses to vegetative propagation pressure then may have resulted in the reduced evenness of the communities under the high vegetative propagule pressure.

Despite vegetative propagule pressure, the growth of the submerged macrophyte communities was greater when the water depth was 30 cm than when it was 70 cm as predicted. The relative low light intensity under deeper water may have limited the growth of the macrophytes [24, 27, 42]. Former studies consistently agree that submerged macrophytes are adverse to live under deeper water [43]. In our study most submerged macrophytes preferred lower water depth (30 cm), yet could still live in water with 70 cm depth, indicating the presence of individual adaptation mechanisms to accommodate different water depths. *H*. *verticillata* and *E*. *nuttallii* effectively absorb the light by extending shoots and forming a canopy on the water [44]. Furthermore, *H*. *verticillata* exhibits a low light compensation point (15 μmol m^-2^ s^-1^) [45]. For *C*. *demersum*, it can grow more leaves for more nutrition because of its advantage in acicular leaf to adapt water depth [46]. *M*. *spicatum* perished in the deeper water (70 cm), possibly resulting from a combination of weak light [47,48] and other factors that we did not know. For instance, the low light under the deep water impairs the uptake phosphate from water of *M*. *spicatum* through shoots and roots, which has negative effects on the growth [49]. These results suggest that water depth should be taken into consideration when restoring submerged macrophyte communities. Artificial measures are needed to increase the establishment chance for submerged macrophyte under comparatively deep water, especially at initial restoration stages.

The evenness of the submerged macrophyte communities increased significantly under deeper water due to biomass reduction of the dominant species (*H*. *verticillata* and *E*. *nuttallii*) and the unchanged biomass production of *C*. *demersum*. Although *M*. *spicatum* perished in deeper water, biomass was substantially less even in lower water depth. Reduced light availability in deeper water [50] produced growth differences among the four submerged macrophytes of communities, thereby altering the community evenness.

We conclude that increasing vegetative propagule pressure under lower water depth can facilitate the establishment success of submerged macrophyte communities. However, if species evenness of submerged macrophyte communities should also be considered during aquatic ecosystem restoration, then vegetative propagule pressure and water depth should be adjusted to a certain level that may not maximize productivity. Further studies should consider the balance between maximizing productivity and maintaining evenness of submerged macrophyte communities.

## Supporting Information

S1 Table Data used from the experiment in analyses. (XLSX)
